# Mouse mutants in schizophrenia risk genes *GRIN2A* and *AKAP11* show EEG abnormalities in common with schizophrenia patients

**DOI:** 10.1038/s41398-023-02393-7

**Published:** 2023-03-13

**Authors:** Linnea E. Herzog, Lei Wang, Eunah Yu, Soonwook Choi, Zohreh Farsi, Bryan J. Song, Jen Q. Pan, Morgan Sheng

**Affiliations:** 1 Stanley Center for Psychiatric Research, Broad Institute of MIT and Harvard, Cambridge, MA USA; 2 Department of Brain and Cognitive Sciences, Massachusetts Institute of Technology, Cambridge, MA USA

**Keywords:** Neuroscience, Schizophrenia, Physiology

## Abstract

Schizophrenia is a heterogeneous psychiatric disorder with a strong genetic basis, whose etiology and pathophysiology remain poorly understood. Exome sequencing studies have uncovered rare, loss-of-function variants that greatly increase risk of schizophrenia [1], including loss-of-function mutations in *GRIN2A* (aka *GluN2A* or *NR2A*, encoding the NMDA receptor subunit 2A) and *AKAP11* (A-Kinase Anchoring Protein 11). *AKAP11* and *GRIN2A* mutations are also associated with bipolar disorder [2], and epilepsy and developmental delay/intellectual disability [1, 3, 4], respectively. Accessible in both humans and rodents, electroencephalogram (EEG) recordings offer a window into brain activity and display abnormal features in schizophrenia patients. Does loss of *Grin2a* or *Akap11* in mice also result in EEG abnormalities? We monitored EEG in heterozygous and homozygous knockout *Grin2a* and *Akap11* mutant mice compared with their wild-type littermates, at 3- and 6-months of age, across the sleep/wake cycle and during auditory stimulation protocols. *Grin2a* and *Akap11* mutants exhibited increased resting gamma power, attenuated auditory steady-state responses (ASSR) at gamma frequencies, and reduced responses to unexpected auditory stimuli during mismatch negativity (MMN) tests. Sleep spindle density was reduced in a gene dose-dependent manner in *Akap11* mutants, whereas *Grin2a* mutants showed increased sleep spindle density. The EEG phenotypes of *Grin2a* and *Akap11* mutant mice show a variety of abnormal features that overlap considerably with human schizophrenia patients, reflecting systems-level changes caused by *Grin2a* and *Akap11* deficiency. These neurophysiologic findings further substantiate *Grin2a* and *Akap11* mutants as genetic models of schizophrenia and identify potential biomarkers for stratification of schizophrenia patients.

## Introduction

A severe psychiatric disorder affecting approximately 0.5% of the global population, schizophrenia is characterized by hallucinations, delusions, disorganized thoughts and behavior, social withdrawal, reduced emotional expression, and cognitive deficits. While genome-wide association studies (GWAS) have identified many genetic loci associated with schizophrenia, it is often difficult to identify the causal gene or to interpret the biological effect of the GWAS common variants, which are mainly non-coding, challenging to fine-map, and associated with only small increases in disease risk (odds ratio typically around 1.1) [5–7]. Compared with GWAS, the rare copy number variants that are associated with schizophrenia confer much higher risk but affect large regions of the genome, making it difficult to identify the relevant pathogenic gene(s) [7, 8]. As a result, the field has been hampered by lack of specific genetic models that can be brought to bear on the mechanisms of schizophrenia.

Recently, the discovery of schizophrenia risk genes has been enhanced by large-scale exome or genome sequencing of tens of thousands of cases versus controls. Such studies have the power to uncover rare loss-of-function coding variants (such as protein-truncating variants, PTVs) that have a large impact on schizophrenia risk [1, 9, 10]. In one of the largest sequencing studies to date of 24,248 cases and 97,322 controls, the Schizophrenia Exome Sequencing Meta-analysis Consortium (SCHEMA) has identified multiple rare loss-of-function genetic variants at exome-wide level of significance that confer substantial disease risk (“SCHEMA genes”; odds ratios in the range of 4-50) [1]. Because these rare variants are often PTVs occurring upstream of the last exon junction, which are predicted to lead to complete loss of function (i.e., null mutations, which presumably lead to haploinsufficiency, rather than missense mutations resulting in a reduced protein function, which presumably do not) [1, 11–14], these disease-causing mutations can be easily modeled by genetic disruption (‘knockout’) in animals such as the mouse. Moreover, these null mutations can be studied in homozygous as well as heterozygous states, the latter being more relevant to the human disease, where only one of the alleles is disrupted in patients. By systematically analyzing the phenotypes of mouse lines bearing loss-of-function mutations in SCHEMA genes (SCHEMA mouse mutants), we hope to discover convergent molecular and neurobiological mechanisms and identify potential biomarker signatures caused by these relatively highly penetrant mutations. Insights derived from these genetic animal models of schizophrenia should further our understanding of the biology of schizophrenia and could aid our efforts to develop more effective treatments [15]. In this study, we characterize by chronic electroencephalogram (EEG) the changes in brain activity caused by mutations in two SCHEMA genes, *GRIN2A* and *AKAP11*.

One of ten genes reaching exome-wide significance [1], *GRIN2A* (glutamate ionotropic receptor NMDA type subunit 2A) is a particularly compelling SCHEMA gene for further study because it is also a significant GWAS hit for schizophrenia [5]. *GRIN2A* encodes the 2A subunit of the N-methyl-D-aspartate (NMDA) receptor (also known as GluN2A or NR2A), a protein that is highly expressed in neurons in the brain and localized at postsynaptic sites of glutamatergic synapses [16, 17]. Reduced NMDA receptor function has long been proposed as a pathophysiologic mechanism underlying schizophrenia, in part because NMDA receptor antagonists at low concentrations can induce psychosis-like symptoms in humans [18]. Interestingly, *GRIN2A* is expressed later in brain development than the genes encoding the GRIN1 and GRIN2B subunits of the NMDA receptor; expression of *GRIN2A* starts postnatally and rises through juvenile and adolescent stages in humans and rodents, inviting comparison with the typical onset of schizophrenia in adolescence and early adulthood [19–21].

*AKAP11* (A-Kinase Anchoring Protein 11) has been identified as a rare-variant large-effect risk gene for schizophrenia, ranking #12 in the recent SCHEMA exome meta-analysis (https://schema.broadinstitute.org/) [1]. In addition, *AKAP11* is a bipolar disorder risk gene, recently identified as the top hit from Bipolar Exome (BipEx) sequencing studies of 13933 bipolar cases and 14422 controls [2]. Thus, *AKAP11* is a shared risk gene for schizophrenia and bipolar disorder, underscoring the genetic overlap between these two disorders on the psychosis spectrum. *AKAP11* (formerly known as *AKAP220*) encodes an A-kinase anchoring protein that is expressed broadly in the body, including in neurons in the brain. Biochemically, AKAP11 interacts with protein kinase A (PKA), a protein that is involved in a variety of biological processes including neuronal plasticity, and with glycogen synthase kinase 3 beta (GSK3β), one of the targets of lithium, a mainstay therapy for bipolar disorder [22–24]. However, AKAP11’s function in the brain and its biological role in psychiatric disease are uncharacterized.

Here we investigate how *GRIN2A* and *AKAP11* loss-of-function affect brain activity by monitoring EEG in mutant mice deficient in these genes. EEG provides a non-invasive, high temporal-resolution, systems-level readout of neural activity in vivo, a neurophysiologic assay that is clinically translatable to human patients. The present work provides an in-depth characterization of EEG phenotypes across different behavioral states (sleep, wake, auditory stimulation) and ages (3 and 6 months) in *Grin2a* and *Akap11* heterozygous (Het) and homozygous knockout (KO) mice, as compared with their wild-type littermates (WT). We found that *Grin2a* and *Akap11* mutant mice possess several EEG features shared with human schizophrenia and bipolar disorder patients, including elevated gamma oscillations at rest [25, 26], attenuated auditory steady-state responses (ASSR) at gamma frequencies [27, 28], and changes in sleep spindle density [29, 30]. These mouse EEG phenotypes reveal systems-level abnormalities caused by (even heterozygous) mutations in *Grin2a* and *Akap11*, and provide further justification, beyond their genetic validity, that these mouse mutants can serve as useful animal models of schizophrenia/bipolar disorder.

## Materials and methods

### Animals

All experiments were approved by the Broad Institute IACUC (Institutional Animal Care and Use Committee) and conducted in accordance with the NIH Guide for the Care and Use of Laboratory Animals. Mice were housed at AAALAC-approved facilities on a 12-hour light/dark cycle, with food and water available *ad libitum*. *Grin2a* (B6;129S-Grin2a < tm1Nak>; RBRC02256, Riken BioResource Center, Saitama, Japan; MGI: 1928506) and Akap11 (B6.Cg-Akap11[tm1.2Jsco/J]; #028922, Jackson Laboratory, Bar Harbor, ME; MGI:5751858) mutant mice on the C57BL/6J background were originally generated as described [31, 32]. *Grin2a* and *Akap11* heterozygous breeding pairs were generated in-house by crossing homozygous KO with C57BL/6J wild-type mice (#000664, Jackson Laboratory). The resulting heterozygous breeding pairs were used to generate *Grin2a* and *Akap11*^−/−^ (homozygous knockout, KO) and ^*+/*−^ (heterozygous, Het) mice and their wild-type (^*+/+*^, WT) littermates. Adult male and female *Grin2a* mice and male *Akap11* mice were used for all experiments (open field testing: *n* = 12 *Grin2a* WT [6 male, 6 female], *n* = 13 *Grin2a* Het [7 male, 6 female], *n* = 11 *Grin2a* KO [5 male, 6 female]; *n* = 11 *Akap11* WT, *n* = 10 *Akap11* Het, *n* = 10 *Akap11* KO; EEG recording: *n* = 12 *Grin2a* WT [6 male, 6 female], *n* = 12 *Grin2a* Het [4 male, 8 female], *n* = 12 *Grin2a* KO [11 male, 1 female]; *n* = 11 *Akap11* WT, *n* = 12 *Akap11* Het, *n* = 12 *Akap11* KO). The sample size (*n~*10–12 mice/group) was estimated based on statistical power calculations for mouse disease models that produce mild phenotypes (e.g., 50% change in EEG power). No randomization was used to allocate animals to experimental groups. 1 male *Grin2a* WT mouse was removed from sleep/wake and MMN analyses at 6 months due to damaged EEG electrodes. Open field behavior and auditory EEG experiments were conducted during the light phase of the daily cycle. All experiments and analyses were conducted by investigators who were blinded to the mouse genotype.

### Open field testing

11- to 14-week-old *Grin2a* and *Akap11* mutant mice and their WT littermates (*n* = 10–13 animals/group) were monitored using the SuperFlex Open Field system (40 cm x 40 cm x 40 cm; Omnitech Electronics, Inc., Columbus, OH) for 60 minutes. The animals’ position was captured in real-time using Fusion system software (Omnitech Electronics, Inc.).

### EEG implantation surgery

6- to 12-week-old mice (*n* = 11–12 mice/group) were deeply anesthetized with isoflurane. A prefabricated EEG/EMG headmount (#8201-SS, Pinnacle Technology, Lawrence, KS) was secured to the skull with four 0.10” intracranial electrode screws (#8403, Pinnacle Technology) at the following stereotactic coordinates: frontal recording electrode (+1.5 AP, 1.5 ML to Bregma), parietal recording electrode (-2 AP, 1.5 ML to Bregma), ground and reference electrodes (bilaterally −1 AP, 2 ML to Lambda). The electromyogram (EMG) electrodes were placed bilaterally in the nuchal muscles. Electrodes were soldered to the EEG/EMG headmount and dental acrylic was used to secure the connections. Animals were given at least one week of post-operative recovery before EEG recording.

### EEG recording

Following recovery from EEG implantation, mice were tethered to the Pinnacle recording system, with at least 3 hours of habituation before testing. Experiments were conducted over a period of 4–5 days. EEG/EMG signals were recorded in freely moving mice across 24 hours of sleep/wake, followed by ASSR and mismatch negativity (MMN) testing (see Supplementary Information for details). Animals remained tethered to the Pinnacle system throughout the testing period with *ad libitum* access to food and water. All signals were digitized at a sampling rate of 1000 Hz, filtered (1–100 Hz bandpass for EEG; 10–1 kHz bandpass for EMG), and acquired using the Sirenia Acquisition program (Pinnacle Technology). EEG recordings took place at two time points, roughly corresponding to 3- and 6-months of age (mouse age at the time of Recording 1: 8–17 weeks, Recording 2: 22-33 weeks). Mice were returned to their home cage between recording sessions.

### EEG analysis

Sleep state classification, power, sleep spindles, ASSR, and MMN were analyzed as described in the Supplementary Information. All analyses shown were conducted using data from the frontal EEG electrode.

### Statistical analysis

All comparisons across genotypes (WT vs. Het vs. KO) were conducted using one-way ANOVAs followed by Tukey post-hoc pairwise comparisons, unless otherwise specified. Variance was similar across groups that were statistically compared.

## Results

### *Grin2a* and *Akap11* mutants exhibit increased and decreased locomotor activity, respectively, in the open field test

**Fig. 1:**
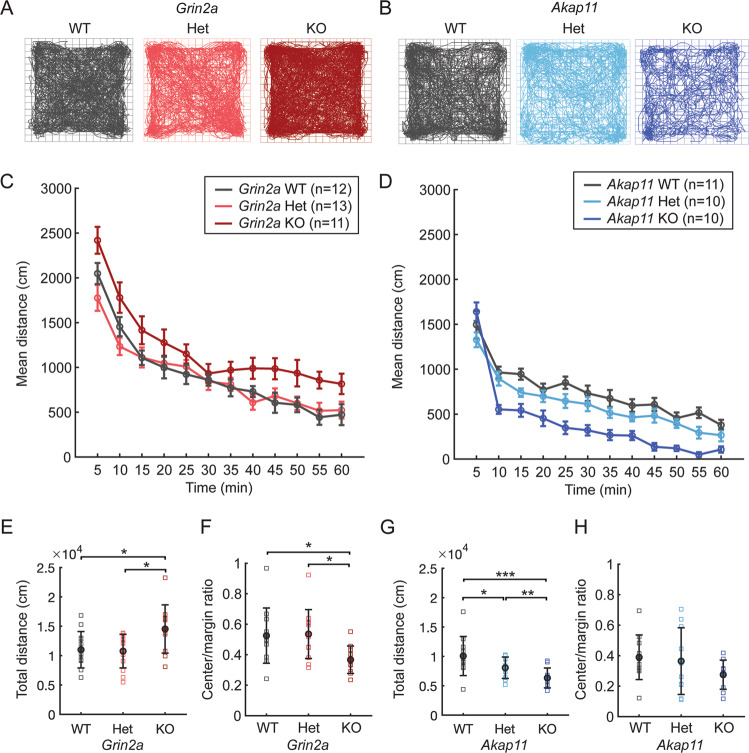
Locomotor behavior of *Grin2a* and *Akap11* mutant mice in open field test. **A**, **B** Representative traces of mouse movements (60-minute test). **C**, **D** Mean distance traveled, binned into 5-minute segments. **E–H** Total distance traveled and ratio of center/margin distance in *Grin2a* (left) and *Akap11* (right) mutants in comparison to WT littermates. Error bars denote mean ± standard error; *p < 0.05, **p < 0.01, ***p < 0.001.

The open-field test is frequently used to measure general locomotor activity and infer anxiety-like behavior in rodents [33, 34]. At 3 months of age, *Grin2a* KO mice showed increased locomotor activity in the open field (Fig. 1A, C, E; ~25% increase from WT; *p* = 0.033), consistent with previous reports [35–37]. *Grin2a*^+/−^ (Het) mice exhibited similar activity levels as WT littermates. In contrast, *Akap11* Het (*p* = 0.0337) and KO (*p* = 7.49e-07) showed reduced locomotor activity ~by 25% and 50%, respectively, compared to their WT littermates (Fig. 1B, D, G), consistent with previous findings [32]. *Grin2a* KO (but not Het) mice had reduced ratios of center/margin distance traveled (by ~30%) relative to WT animals (*p* = 0.0438), suggesting anxiety-like behavior (Fig. 1F). *Akap11* Het and KO had similar center/margin ratios as WT littermates (Fig. 1H).

### *Akap11*^−/−^ mice exhibit NREM sleep deficits

**Fig. 2:**
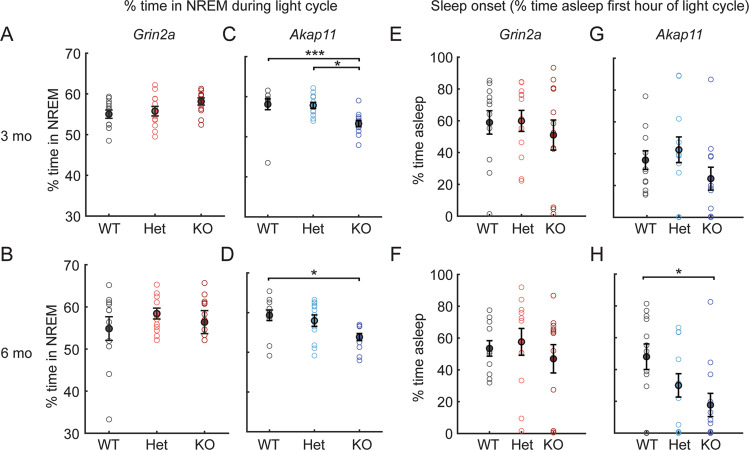
NREM sleep and sleep onset time of *Grin2a* and *Akap11* mutant mice. **A**–**D** % of time spent in NREM sleep during the light cycle in 3- and 6-month *Grin2a* and *Akap11* mutants. **E**–**H** Sleep onset, as quantified by the % of time spent asleep in the first hour of the light cycle. Error bars denote mean ± standard error; *p* < 0.05, **p < 0.01, ***p < 0.001; *n* = 11–12 mice/group.

Patients with schizophrenia often show sleep abnormalities, including reduced sleep, increased sleep, delayed sleep onset, or fragmented sleep [38, 39]; altered sleep patterns (e.g., more sleep in depressive phase, less sleep in manic phase) are also found in bipolar disorder [40]. To assess sleep differences in *Grin2a* and *Akap11* mutant mice, we recorded EEG/EMG signals across 24 hours and used a feature-based model (see Supplementary Information) to classify periods of NREM, REM, and wake. Sleep analyses were conducted during the light cycle when mice are predominantly asleep. *Grin2a* heterozygous and homozygous mutant mice exhibited similar sleep patterns as their WT littermates (Fig. 2A–B). *Akap11*^*-/-*^ mice exhibited reduced (~10%) NREM sleep relative to WT littermates at 3 months (*p* = 0.0052) and 6 months of age (*p* = 0.0106), whereas *Akap11*^*+/*−^ animals were not significantly different than WT (Fig. 2C,D). At 6-months of age, *Akap11*^−/−^ mice also showed delayed sleep onset at the start of the light cycle, relative to WT littermates (*p* = 0.0207, Fig. 2E–H). Neither *Grin2a* or *Akap11* mutants (heterozygous or homozygous) displayed abnormal sleep fragmentation (Figure S1), although NREM bout length was reduced in *Akap11* KO (*p* = 0.0478) compared with WT littermates (Figure S1G).

### Elevated resting gamma power in *Grin2a* and *Akap11* mutant mice

**Fig. 3:**
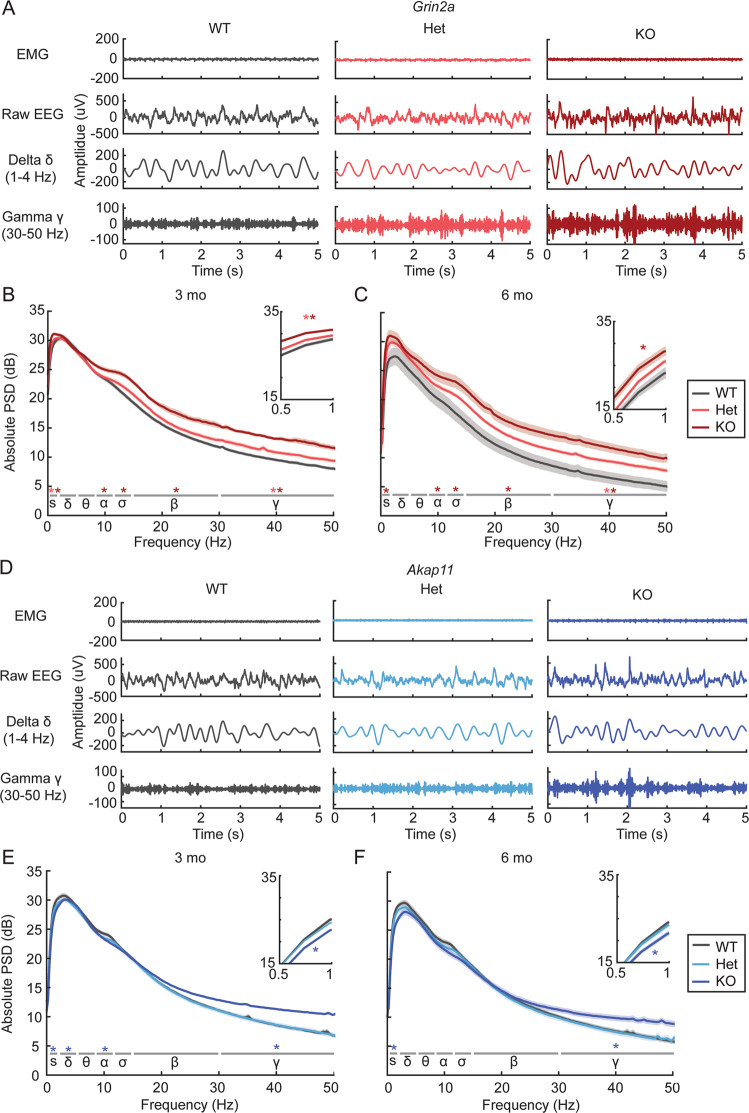
Power spectral analysis of brain oscillations in *Grin2a* and *Akap11* mutant mice during NREM sleep. **A** Representative EMG and EEG (raw, delta- and gamma-filtered) traces from *Grin2a* WT, Het, and KO animals during NREM sleep. **B**, **C** Absolute NREM power spectra for 3- and 6-month *Grin2a* mutants during the light cycle. Light and dark red/blue asterisks indicate oscillations for which ^+/−^ and ^−/−^ mice, respectively, differed significantly from WT littermates (*p* < 0.05) in each of the frequency bands: slow (s), 0.5–1 Hz; delta (δ), 1–4 Hz; theta (θ), 4–8 Hz; alpha (α), 8–12 Hz; sigma (σ), 12–15 Hz; beta (β), 15–30 Hz; gamma (γ), 30–50 Hz. Shading indicates mean ± standard error. Insets show magnified view of the graph in the slow oscillation range (0.5–1 Hz). *n* = 11–12 mice/group. **D** Representative EMG and EEG traces from *Akap11* WT, Het and KO animals during NREM sleep. **E**, **F** Absolute NREM power spectra for 3- and 6-month *Akap11* mutants during the light cycle.

Many patients with schizophrenia exhibit altered EEG oscillatory power at rest (e.g., during sleep or in the absence of a task) especially increased gamma oscillations [41, 42]. Focusing our analysis on NREM sleep, where EEG signals are less prone to movement-related variation, we measured absolute power of brain oscillations in *Grin2a* and *Akap11* mutants versus their WT littermates for each frequency band (slow: 0.5–1 Hz, delta: 1–4 Hz, alpha: 8–12 Hz, sigma: 12–15 Hz, beta: 15–30 Hz, gamma: 30–50 Hz). *Grin2a*^*+/*−^ and ^−*/*−^ mice exhibited gene dose-dependent increases in gamma oscillations (Fig. 3A–C, S2): a 10% and ~20% increase in absolute gamma power compared with WT for heterozygous and homozygous mutants, respectively (Het, 3 months: *p* = 0.0187; Het, 6 months: *p* = 0.0385; KO, 3 months: *p* = 1.07e−08; KO, 6 months: *p* = 1.15e-04). Resting gamma power was also increased by ~10% in *Akap11*^−*/*−^ mice at 3 months (*p* = 1.80e-04) and 6 months (*p* = 0.0112) of age, but unlike *Grin2a* heterozygotes, *Akap11* heterozygous mutants were not significantly different from WT (Fig. 3D–F, S2). Most observed increases in resting gamma power were associated with increased spectral entropy and/or a reduced aperiodic exponent (“flatter” PSD) in the parameterized power spectrum (Table S1). Besides NREM, elevated gamma power was also found during REM sleep (Figure S3, S4) and quiet wake (Figure S5) in *Grin2a* and *Akap11* homozygous mutants. *Grin2a* (but not *Akap11*) heterozygous mutants exhibited an intermediate phenotype of increased gamma oscillations during REM sleep (Figure S3), although this result did not reach statistical significance (3 months: *p* = 0.190; 6 months: *p* = 0.111)

We found changes in oscillation power outside of gamma frequencies. The EEGs of *Grin2a* KO exhibited increased power broadly across multiple frequency bands, including slow oscillations (3 months: *p* = 8.12e−08; 6 months: *p* = 0.0023), alpha (3 months: *p* = 0.0252; 6 months: *p* = 0.0357), sigma (3 months: *p* = 4.00e−05; 6 months: *p* = 0.0030), and beta (3 months: *p* = 2.71e−06; 6 months: *p* = 7.76e−04) (Fig. 3B, C; Fig. S2). *Grin2a* Hets also exhibited increased power of slow oscillations at 3 months (Figure S2; *p* = 0.0259). Unlike *Grin2a* KO mice, *Akap11*^*−/−*^ animals had reduced power of slow oscillations (Fig. 3E, F, S2), by ~15% compared to WT (3 months: *p* = 2.85e−05; 6 months: *p* = 0.0059), as well as reduced delta (*p* = 0.0191) and alpha (*p* = 0.0375) oscillations at 3 months (Figs. 3E, S2). Heterozygous *Akap11* mutants showed no significant changes in oscillation power for any frequency band.

### *Grin2a* and *Akap11* mutants exhibit gene dose-dependent increases and decreases in sleep spindles, respectively

**Fig. 4:**
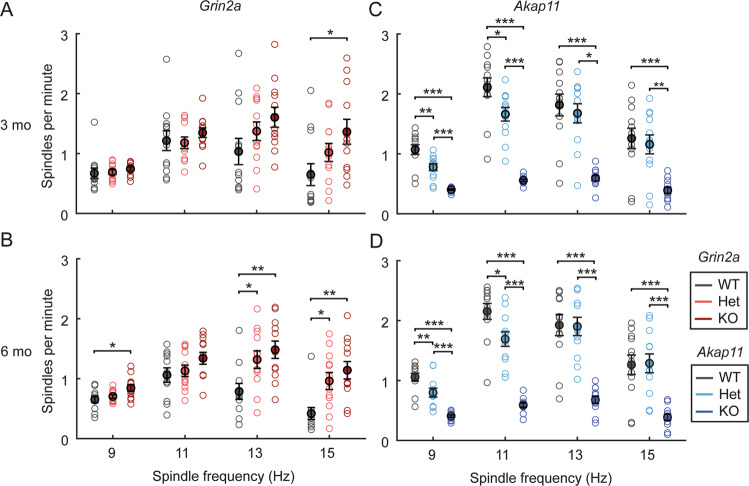
Sleep spindle density in *Grin2a* and *Akap11* mutants. **A–D** Density for 9 Hz, 11 Hz, 13 Hz, and 15 Hz spindles during NREM sleep during the light cycle. Error bars show mean ± standard error; *p* < 0.05, **p < 0.01, ***p < 0.001; *n* = 11–12 mice/group.

Sleep spindles, an EEG feature possibly linked to overnight memory consolidation, are commonly reduced in schizophrenia patients [30, 43, 44]. Measuring sleep spindle density during NREM sleep in the light cycle, we found striking gene dose-dependent increases in sleep spindle density in *Grin2a* mutants, especially at 6 months (Fig. 4A, B; ~10-40% increase in heterozygous, and 25-50% increase in homozygous mutants). This increase in spindle density occurred across a range of spindle frequencies from 9 Hz to 15 Hz (3 months, KO, 15 Hz: *p* = 0.0234; 6 months, Het, 13 Hz: *p* = 0.0340; 6 months, Het, 15 Hz: *p* = 0.0192; 6 months, KO, 9 Hz: *p* = 0.0188; 6 months, KO, 13 Hz: *p* = 0.0044; 6 months, KO, 15 Hz: *p* = 0.0016). In contrast, *Akap11* mutants showed the opposite phenotype of decreased spindles (Fig. 4C, D), also in a gene dose-dependent manner (heterozygotes ~25% reduction and homozygous KO ~50–75% reduction, compared with WT littermates) (3 months, Het, 9 Hz: *p* = 0.0029, 3 months, Het, 11 Hz: *p* = 0.0193; 3 months, KO, 9 Hz: *p* = 5.53e−09; 3 months, KO, 11 Hz: *p* = 6.58e−11; 3 months, KO, 13 Hz: *p* = 2.27e−06; 3 months, KO, 15 Hz: *p* = 2.61e−04; 6 months, Het, 9 Hz: *p* = 0.0088; 6 months, Het, 11 Hz: *p* = 0.0106; 6 months, KO, 9 Hz: *p* = 1.03e−08; 6 months, KO, 11 Hz: *p* = 1.39e−11; 6 months, KO, 13 Hz: *p* = 1.00e−06; 6 months, KO, 15 Hz: *p* = 1.52e−04).

### *Grin2a* mutants exhibit attenuated ASSR at gamma frequencies

**Fig. 5:**
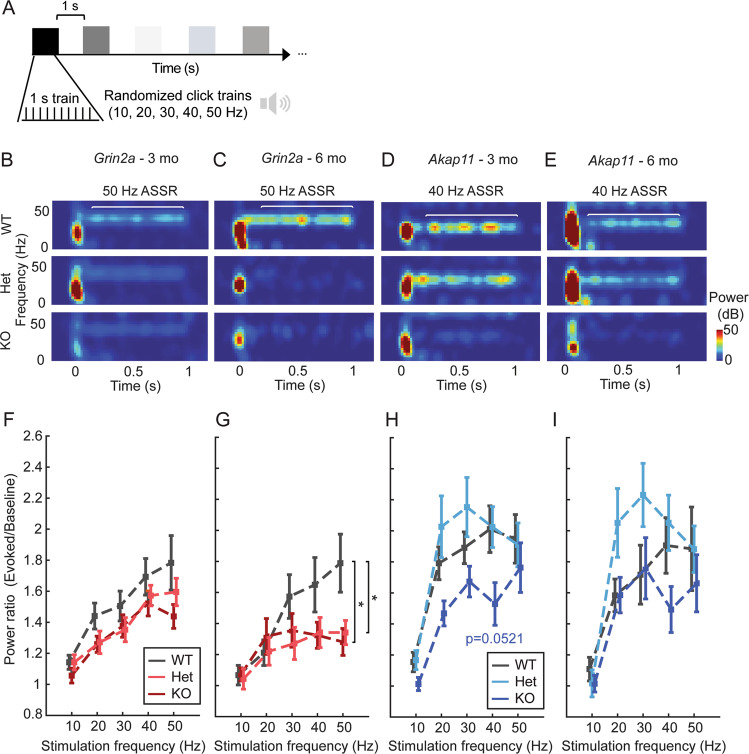
Auditory steady-state responses (ASSR) in *Grin2a* and *Akap11* mutants. **A** Schematic of ASSR paradigm. **B**–**E** Representative ASSR for 3- and 6-month-old *Grin2a* and *Akap11* animals. **F**–**I** Power ratio quantification of the evoked (0.2–1 s post-stimulus) / baseline (0.5 s pre-stimulus) response for 10-50 Hz auditory stimulation (evaluated using Welch’s method, window = 0.5 s, overlap = 0.25 s). Asterisks indicate significant differences in Het or KO vs. WT littermates (*p* < 0.05). Error bars denote mean ± standard error.

ASSR is a highly translatable sensory processing assay that measures cortical entrainment by comparing the evoked response to click trains of different frequencies relative to pre-stimulus activity [45]. ASSR is typically reduced for gamma (40–50 Hz), but not lower frequency (<40 Hz), stimuli in schizophrenia and bipolar disorder patients relative to healthy controls, signifying impaired entrainment of cortical gamma rhythms [25, 27, 28, 45, 46]. Using an ASSR paradigm with trains of randomized 10-50 Hz stimulation frequencies (Fig. 5A), we found a significant reduction of 50 Hz entrainment (as quantified by the power ratio) for 6-month-old Grin2a Hets and KO mice, which was not present at 3 months (Fig. 5B–G). Relative to WT, homozygous (*p* = 0.0467) and heterozygous (*p* = 0.0214) *Grin2a* mutants showed a ~30% reduction in 50 Hz ASSR at 6 months (Fig. 5C, G). There was also a trend (*p* = 0.0521) towards decreased 40 Hz ASSR for *Akap11* KO at 3 months (Fig. 5H), but no other consistent differences between *Akap11* mutants and WT (Fig. 5D, E, H, I).

### *Grin2a*^−/−^ and *Akap11*^−/−^ mice show reduced responses to deviant auditory stimuli in the MMN paradigm

**Fig. 6:**
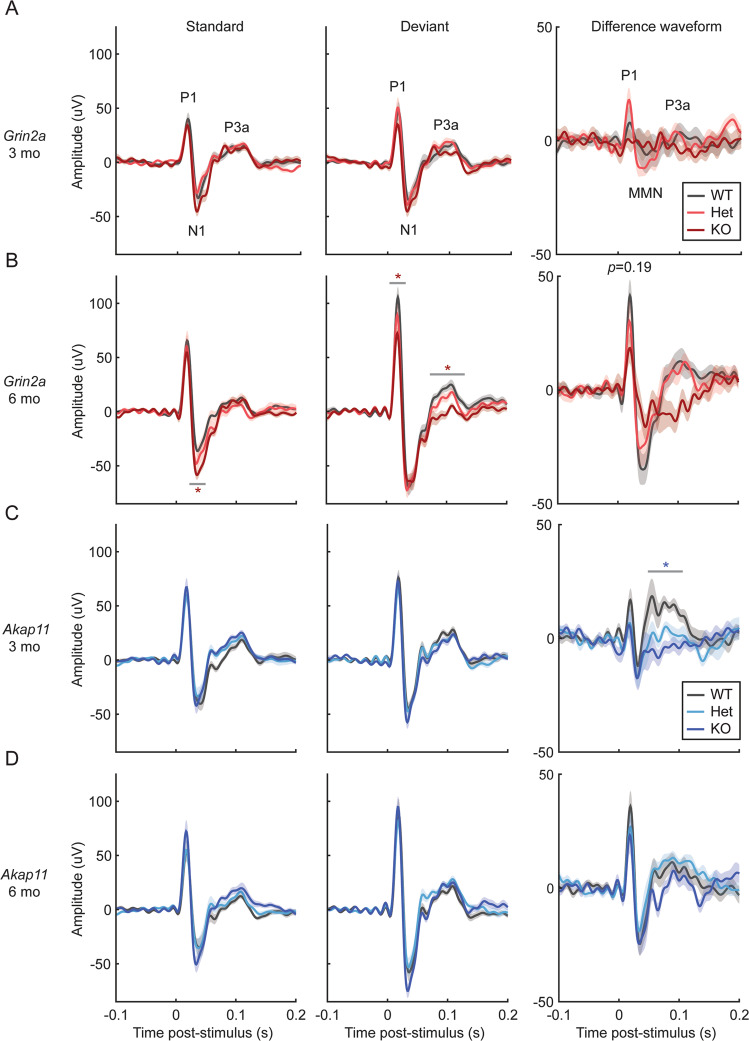
Mismatch negativity (MMN) responses in *Grin2a* and *Akap11* mutants. **A**–**D** Mean response to standard (*n* = 900 trials), deviant (*n* = 100 trials) and difference waveforms [response to deviant tones minus the response to standard tones], averaged across each genotype. Shading indicates mean ± standard error. Red or blue asterisks indicate statistical differences in peak amplitude between WT and KO animals, which was assessed for the P1, N1/MMN, and P3a peaks (as indicated on the first graphs). *p* < 0.05, **p < 0.01, ***p < 0.001; *n* = 11–12 mice/group.

MMN (sometimes known as the “auditory oddball” paradigm) measures novelty-related responses to unexpected stimuli by comparing the event-related potentials in response to infrequent stimuli (deviant, 10% occurrence) vs. frequent stimuli (standard, 90% occurrence) that differ by at least one parameter (e.g., frequency, duration, or amplitude) [47, 48]. Schizophrenia patients display reduced responses to unexpected auditory stimuli, as reflected by the decreased amplitude of event-related potential components MMN and P3a, compared with healthy comparison subjects [47, 49, 50]. These differential responses are thought to reflect deficits in sensory discrimination and novelty-related attention shifts, respectively. We found that *Grin2a* and *Akap11* KO exhibited altered responses to auditory stimuli during the MMN paradigm (Fig. 6). *Grin2a*^−/−^ animals at 6 months (but not 3 months) exhibited reduced P1 (*p* = 0.031) and reduced P3a (*p* = 0.0099) peak amplitudes (~30% and 40% decrease from WT levels, respectively) in response to deviant tones, while the N1 peak was increased in response to standard tones in these animals (~35% increase from WT level, *p* = 0.0217) (Fig. 6A-B, S6A-C). P1 and MMN amplitudes in the difference waveform (response to deviant tones minus the response to standard tones) of 6-month-old *Grin2a* KO mice were reduced compared to WT and Het (Fig. 6B), though these results were not statistically significant (P1: *p* = 0.19; MMN: *p* = 0.93) due to large variation in the data. *Akap11*^−/−^ animals showed a strongly reduced P3a amplitude in the difference waveform at 3 months (~70% reduction vs. WT; *p* = 0.0192), while *Akap11* heterozygous mutants had similar responses as their WT littermates (Fig. 6C, D, S6).

## Discussion

Recent large-scale exome-sequencing efforts have uncovered rare genetic variants that confer a high risk for schizophrenia, including *GRIN2A* and *AKAP11* [1, 2]. These relatively penetrant, loss-of-function mutations lend themselves to disease modeling in genetically modified mice. Besides their genetic validity, do *Grin2a* and *Akap11* mutant mice have any neurobiological phenotype that resembles the human disease (schizophrenia and/or bipolar disorder)? Using a neurophysiological assay (EEG) that is largely translatable between rodents and humans, we report that *Grin2a* and *Akap11* mutants show several key EEG abnormalities that are also observed in patients with schizophrenia and bipolar disorder (summarized in Table S2), including increased resting gamma power and reduced evoked gamma oscillations, altered sleep spindle density, and abnormal MMN (in particular, altered P3a event-related potentials in the MMN paradigm). These shared EEG abnormalities suggest that *Grin2a* and *Akap11* deficiencies impact, at least in part, the same brain networks and circuits that go awry in schizophrenia patients. One limitation of our study design is that male and female *Grin2a* mutant mice, but only male *Akap11* mutant mice, were used; therefore, we cannot exclude the possibility that female *Akap11* mutant mice may exhibit a different phenotype than the male *Akap11* mutants tested.

### Gamma oscillations

A major finding of our study is that *Grin2a* and *Akap11* mutants show elevated gamma power during sleep and quiet wake (Figs. 3, S3, S5), consistent with EEG findings in schizophrenia [25, 41, 42, 51] and to a lesser extent, bipolar disorder patients [51–53]. Gamma oscillations are generated by local cortical networks of fast-spiking, parvalbumin-positive (PV^+^) interneurons and excitatory pyramidal cells [54, 55]. Evidence of deficiency of PV^+^ interneuron function has been found in postmortem studies of schizophrenia patient brains [26, 56, 57], and resting gamma power is elevated in NMDAR hypofunction rodent models [58, 59], including mice lacking NMDAR transmission specifically in PV^+^ interneurons [60]. In the present study, *Grin2a* and *Akap11* mutants showed increased spectral entropy and/or a reduced aperiodic exponent (indicative of a “flatter” PSD) in the parameterized power spectrum during NREM sleep (Table S1), indicating that the resting gamma increases observed in *Grin2a* and *Akap11* mutants (Fig. 3) most likely reflect increases in asynchronous firing that manifest as increased broadband gamma [61, 62]. These increases in asynchronous neural activity may be driven by an enhanced E/I ratio [63], potentially arising from insufficient PV inhibition, as has been reported for PV-Cre/NR1 f/f mice [62]. In contrast to elevated resting gamma power, evoked gamma oscillations such as ASSR are reduced in schizophrenia [25, 27, 45, 46, 64] and bipolar disorder patients [28, 65, 66]. Similarly, we observed reduced 50 Hz ASSR in both *Grin2a* KO and Hets at 6 months and a strong trend towards reduced 40 Hz ASSR in *Akap11* KO at 3 months (Fig. 5).

Our findings are in line with the long-standing “glutamatergic hypothesis” that NMDAR dysfunction may be an underlying cause of schizophrenia [18, 67]. Indeed, NMDA receptors, including GRIN2A, are important for the function of PV^+^ interneurons [68], and undergo changes during development that may make these cells particularly sensitive to perturbation in psychiatric disease [69]. Importantly, even in the heterozygous state (which is the most relevant to human schizophrenia [1]), *Grin2a* mutations had a significant effect on resting (particularly during NREM sleep) (Fig. 3C) and stimulus-evoked gamma power (Fig. 5G). Overall, our findings suggest that *Grin2a* and *Akap11* deficiency affect convergent neural pathways, potentially involving circuits of PV^+^ interneurons and excitatory neurons, that drive gamma oscillations [55].

In our study, *Grin2a*^−/−^ mice exhibited increases in spontaneous power across multiple frequency bands (Fig. 3), reminiscent of an “over-excitation” phenotype [70]. In contrast, broadband power increases (other than gamma) were not observed in *Akap11* mutants, and slow oscillations were reduced. Intriguingly, mutations in *GRIN2A*, but not *AKAP11*, are associated with certain epilepsy disorders (including epilepsy-aphasia syndromes [1, 3] and severe developmental and epileptic encephalopathy [71]), and a drug that acts as a positive allosteric modulator of GRIN2A-containing NMDA receptors (GNE-0723) reduces spontaneous power (including slow oscillation power) in mouse models of Dravet syndrome and Alzheimer’s disease [35, 72]. These and our findings further substantiate a link between schizophrenia and epilepsy [73], which is affected by *GRIN2A*, but not *AKAP11* mutation [1, 2].

### Sleep spindles

Sleep spindles (12-15 Hz) are another EEG feature typical of NREM sleep, generated by neurons across the cortex, thalamus, and thalamic reticular nucleus (TRN) [29]. Although rather heterogeneous, patients with schizophrenia or bipolar disorder have on average a lower sleep spindle density relative to healthy comparison subjects [30, 40, 74]. Despite sharing several other key features, the EEG phenotypes of *Grin2a* and *Akap11* mutants showed *opposite* and striking changes in sleep spindles. *Grin2a* mutants had elevated sleep spindle density, whereas *Akap11* mutants showed fewer spindles (Fig. 4). In this context, it is noteworthy that both *Grin2a* and *Akap11* heterozygous mutants exhibited a sleep spindle phenotype intermediate between homozygous knockouts and wild-type littermates. Because *Grin2a* and *Akap11* mutations have such disparate effects on spindle density, it is tempting to speculate that the heterogeneity of spindle density in human patients might arise in part from underlying genetic heterogeneity. The opposite effects of *Grin2a* and *Akap11* on sleep spindles implies that these genes differentially regulate the thalamocortical circuits responsible for spindle generation [44]. Although the precise neurophysiologic mechanisms remain to be discovered, our findings suggest that sleep spindles could serve as a biomarker separating different circuit mechanisms affected by *Grin2a* and *Akap11* loss-of-function and be potentially useful in stratifying the human disease population.

### Mismatch negativity (MMN)

The MMN test is a clinically translatable assay of sensory processing of familiar versus unexpected stimuli [47, 75]. The auditory event-related potential peak components P1, N1, and P3a are thought to represent early detection, automatic sensory discrimination, and novelty-related attention shifts, respectively [76]. On average, schizophrenia [49, 77, 78] and bipolar disorder [79, 80] patients tend to exhibit reduced N1 and P3a peak amplitudes, typically measured in the difference waveform (the responses to deviant stimuli minus the responses to standard stimuli). Interestingly, *Grin2a*^−/−^ (6 months) and *Akap11*^−/−^ mice (3 months) exhibited reduced P3a responses to deviant stimuli (Fig. 6). The N1/MMN peak in the difference waveform is reported to be reduced on average in schizophrenia and bipolar disorder patients; however, we found no significant changes in *Grin2a* or *Akap11* mutant mice, perhaps because of high variability in this metric [76, 77, 81]. 6-month-old *Grin2a*^−/−^ mice exhibited increased N1 amplitude in response to standard tones (Fig. 6B), potentially indicating lack of habituation to the repeated stimulus.

## Conclusion

The convergent and divergent EEG features identified in this study highlight systems-level functional changes in the brain which may provide insight into the neurobiological pathways affected by the loss of *Grin2a* and *Akap11*. Overall, the considerable overlap in EEG phenotypes between mouse mutants and human patients gives further credence to *Akap11*- and *Grin2a*-deficient mice as animal models of schizophrenia, particularly so as heterozygous mutants—SCHEMA mutations are found as heterozygotes in humans—tend to show abnormal phenotypes that are intermediate between knockout and WT (e.g., *Grin2a*^+/−^ in gamma oscillation; *Grin2a*^+/−^ and *Akap11*^+/−^ in spindle density). Further investigation of the mechanistic foundations for these EEG changes, including transcriptomic and proteomic characterizations of *Grin2a* and *Akap11* mutants, will help elucidate which brain regions, cell types, and molecular pathways contribute to the network-level dysfunctions of schizophrenia and bipolar disorder.

## Supplementary information

Supplemental Information

## Data Availability

LUNA software (http://zzz.bwh.harvard.edu/luna, v0.26) was used for sleep/wake EEG analysis. The Tort lab’s script (https://github.com/tortlab/phase-amplitude-coupling, v1.0) was used for phase-coupling analysis, and the FOOOF algorithm (https://fooof-tools.github.io/fooof/index.html, v1.0.0) was used for spectral parameterization. All other analyses were conducted using custom Python and MATLAB (MathWorks, Natick, MA, RRID: SCR_001622) scripts, which the authors will provide upon request.
