# Supplementary material for: Mouse mutants in schizophrenia risk genes GRIN2A and AKAP11 show EEG abnormalities in common with schizophrenia patients
Source: Transl Psychiatry. 2023 Mar 13;13:92. doi: 10.1038/s41398-023-02393-7 (PMC10011509; doi:10.1038/s41398-023-02393-7)
Supplement: Supplementary file 1 — Supplemental Information [file 41398_2023_2393_MOESM1_ESM.docx]

**Mouse mutants in schizophrenia risk genes *GRIN2A* and *AKAP11* show EEG abnormalities in common with schizophrenia patients**

Linnea E. Herzog^a,1^, Lei Wang^a^, Eunah Yu^a^, Soonwook Choi^a,b^, Zohreh Farsi^a^, Bryan Song^a^, Jen Q. Pan^a^, Morgan Sheng^a,b,1^

^a^ Stanley Center for Psychiatric Research, Broad Institute of MIT and Harvard, Cambridge, MA, USA

^b^ Department of Brain and Cognitive Sciences, Massachusetts Institute of Technology, Cambridge, MA, USA

Cognitive Sciences, Massachusetts Institute of Technology, Cambridge, MA, USA

**SUPPLEMENTAL MATERIALS AND METHODS**

**EEG recording**

Sleep/wake: Signals were recorded for 24 hours from the onset of the dark phase, ZT12 (6 pm EDT or 7 pm EST).

Auditory steady-state responses (ASSR): MATLAB was used to generate 1 s auditory click trains of 10-50 Hz through the audio speaker (GTO629, JBL by Harman) at ~70 dB. A CompactDAQ system (cDAQ-9171, National Instruments) was used to generate a TTL pulse with each click train sent to Sirenia software to determine stimulus timing throughout the test. 20 randomized trials of each stimulus (10-50 Hz) were delivered for a total of 100 trials lasting 25 minutes.

Mismatch negativity (MMN): MATLAB was used to generate 100 ms tones of either 6000 or 8000 Hz through the audio speaker (GTO629, JBL by Harman) at ~70 dB. A CompactDAQ system (cDAQ-9171, National Instruments) was used to generate a TTL pulse with each click train that was sent to Sirenia software to determine stimulus timing throughout the test. Each test consisted of 1000 randomized trials of 900 standard (6000 Hz) stimuli and 100 deviant (8000 Hz) stimuli. To control for animals’ differential response to pitch, a “flip-flop control” test (using 8000 Hz standard and 6000 Hz deviant tones) was conducted on a different day, and the response to the same tone (8000 Hz) as deviant vs. standard was compared.

**EEG analysis**

Sleep state classification and power: Sleep state classification (NREM, REM or Wake) was performed on 10 s epochs of EEG/EMG data using a machine learning model developed by LW. The model is developed using the light gradient boosting machine (LightGBM), which is an ensemble model based on the decision tree, built using well-established EEG/EMG hallmarks of behavioral state (e.g., EMG amplitude, delta oscillation amplitude, delta/theta ratio). The LightGBM model achieves an overall accuracy of 95.2% and a Cohen’s kappa of 0.91 (a measurement of the inter-rater reliability that accounts for the agreement rate by chance; kappa values greater than 0.8 are considered as nearly perfectly in agreement [1]), which is on par with the performance of human experts reading the EEG traces manually. Wake and NREM had higher f1-scores (the harmonic mean of precision and recall) than REM; wake = 0.96, NREM = 0.95, and REM = 0.85. LUNA software for EEG analysis (http://zzz.bwh.harvard.edu/luna) was used to compute stage duration, sleep onset (defined as % time asleep during the first hour of the light cycle), bout length and number, absolute and relative power for each band (slow: 0.5-1 Hz, delta: 1-4 Hz, theta: 4-8 Hz, alpha: 8-12 Hz, sigma: 12-15 Hz, beta: 15-30 Hz, gamma: 30-50 Hz) separately for the light and dark cycle. Measurements taken during NREM/REM sleep were analyzed during the light cycle, where mice are predominantly asleep. One 3-month-old WT *Grin2a* animal was excluded from NREM/REM power analyses due to excessive line noise during the light cycle. Measurements taken during wake were analyzed during the dark cycle, where mice are predominantly awake. Periods of quiet wake were defined as 10 s epochs where the mean EMG amplitude during wake did not exceed 3 standard deviations above or below the mean EMG amplitude during NREM sleep (the mouse is presumed to be immobile).

Sleep spindles: Sleep spindles were detected as described previously [2]. Briefly, spindles were defined as intervals for which the wavelet power exceeded a threshold of a) 6 times the stage-specific median for that mouse for at least 0.3 seconds (the spindle ‘core’), and b) 3 times the stage-specific median for at least 0.5 seconds (the ‘flanking’ waxing/waning region), but not more than 3.0 seconds in total. The threshold of 6 was determined by applying Otsu’s method for selecting a threshold that maximizes the between-class variance in wavelet power between putative “spindle” and “non-spindle” intervals, with the flanking threshold being 50% of the core threshold (i.e., 3). Spindles within 0.5 seconds of each other were merged, unless the above-mentioned 3.0 second criterion would be violated, in which case they were both excluded. We found that defining thresholds as a multiplicative function of the individual’s median wavelet power was more robust compared to using the mean, as wavelet power has a very skewed distribution and outliers (i.e., in large part reflecting true spindles) disproportionately influence the mean but not the median. Four target spindle frequencies (FC=9, 11, 13, 15 Hz) were chosen to include both slow (9-11 Hz) and fast (13-15 Hz) spindles, which are associated with frontal and centro-parietal sources, respectively [3]. Each analysis detected spindles with a peak frequency approximately ± 1 Hz. For example, “FC=11 Hz spindles,” the group of spindles detected when targeting a center frequency of 11 Hz, is likely to encompass a range of spindle frequencies ranging mostly between 10 and 12 Hz. The maximum number of spindles was found in FC=11 Hz spindles across genotypes, consistent with previous reports that mouse spindle frequencies are centered between 10-12 Hz [2, 4].

Auditory steady-state responses (ASSR): ASSR were analyzed using custom MATLAB scripts to quantify the evoked response to different frequency auditory stimuli [5]. For each randomized stimulation frequency (10-50 Hz), trials were segmented into 2 s windows (0.5 s before stimulus onset to 1.5 s after stimulus onset) as detected from the TTL pulse. Successive trials of the same stimulus were removed from the analysis, as well as large-amplitude (>1800 uV) trials containing movement-related artifacts. EEG power was evaluated corresponding to the stimulus frequency (10-50 Hz) using Welch’s method (*pwelch* algorithm, 0.5 s window, 0.25 s overlap) during the baseline (0.5 s before stimulus onset) and stimulus-evoked (0.2 to 1 s following stimulus onset) periods. The entrainment for each stimulation frequency was quantified using the average power ratio (evoked/baseline power). Representative trial-averaged Z-scored power spectrograms were plotted using the spectrogram algorithm.

Mismatch negativity (MMN): The peak components of event-related potentials (ERPs) generated from the MMN test (P1, N1/MMN, and P3a) were analyzed using custom MATLAB scripts [6]. P1 refers to the first positive peak of the ERP. N1 and MMN both refer to the first negative peak of the ERP (deemed N1 in the response to standard or deviant tones, and MMN in the difference waveform). P3a refers to the second positive peak in the ERP. Trials were segmented into 1 s windows (0.4 s before stimulus onset to 0.6 s after stimulus onset) as detected from the TTL pulse. Trials were normalized to the baseline response (average voltage across the trial) and large-amplitude (>1800 uV) trials containing movement artifacts were removed from the analysis. The average evoked potentials from standard trials preceding the deviant, deviant trials, and the difference waveform (deviant-standard) were plotted for each genotype for visualization. The peak amplitude of the P1 (maximum peak detected 0-0.05 s post-stimulus), N1/MMN (minimum peak detected 0-0.1 s post-stimulus) and P3a (maximum peak detected 0.05-0.15 s post-stimulus) components were calculated for each animal.

Age x group interactions: To assess whether there were interactions between the age x group, we conducted two-way ANOVAs on the main results that included both 3- and 6-month timepoints, using age and genotype as the independent variables and the result as the dependent variable.

Cross-frequency coupling: To determine whether the increases in gamma power found in *Grin2a* and *Akap11* mutant mice during NREM sleep are related to up/down states of the slow oscillation, we compared the cross-frequency coupling between delta phase and gamma amplitude in the WT, Het and KO groups. Delta/gamma cross-frequency coupling was estimated by the modulation index (MI) [7-9]. Comodulation maps were constructed using the Hilbert transform to calculate the MI at phase frequencies from 0.5 to 6 Hz (1 Hz step) and amplitude frequencies from 30 to 150 Hz (5 Hz step). Shannon entropy of the distribution of mean amplitudes per phase (divided into 18 bins) in each frequency bin was calculated to obtain the cross-frequency MI for periods of NREM sleep during the light cycle. The MI between delta oscillations (1-4 Hz) and low gamma band (30-50 Hz) was calculated for group comparisons [7].

Spectral entropy and parametrization: To address whether the increase in gamma power is due to synchronous or asynchronous activity, the spectral entropy of relative power in the gamma range (30-50 Hz) was compared between *Grin2a* and *Akap11* WT, Het, and KO during NREM sleep. The entropy of the distribution (in bits) was calculated based on information theory [9]:


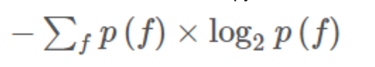


where p(f) is the normalized power, f corresponds to each frequency bin. Higher entropy values indicate more uniform power distributions, consistent with asynchronous activity or broadband noise, while lower entropy values are consistent with power centered around a specific peak (e.g. 40 Hz gamma).

To further analyze the nature of gamma power increases, the FOOOF (fitting oscillations & one over f) algorithm (version 1.0.0) was used to parameterize power spectra into periodic and aperiodic components, which reflect synchronous or asynchronous activity, respectively [10]. Settings for the algorithm were set as: peak width limits: (0.5, 12.0); max number of peaks: inf; minimum peak height: 0.0; peak threshold: 2.0; and aperiodic mode: 'fixed'. Power spectra were parameterized across the frequency range 30.0 to 50.0 Hz with a frequency resolution of 0.25 Hz. The aperiodic exponent and periodic power of the largest peak were compared across *Grin2a* and *Akap11* WT, Het, and KO.

**SUPPLEMENTAL RESULTS**

Age x group interactions: We found no significant interactions between the age x group for any of the tests conducted. For Grin2a mice, these included slow (*p*=0.5879), alpha (*p*=0.9158), sigma (*p*=0.8743), beta (*p*=0.8967) and gamma (*p*=0.9317) power during NREM sleep; 9 Hz (*p*=0.5408), 13 Hz (*p*=0.9390) and 15 Hz (*p*=0.8696) sleep spindles; 50 Hz ASSR (*p*=0.5785; N1 amplitude in the standard waveform (*p*=0.2806), P1 (*p*=0.3706) and P3a (*p*=0.2091) amplitude in the deviant waveform during mismatch negativity. For Akap11 mice, these included NREM stage duration (*p*=0.8882) and sleep onset (*p*=0.2254); slow (*p*=0.8895), delta (*p*=0.9502), alpha (*p*=0.8083), and gamma (*p*=0.8409) power during NREM sleep; 9 Hz (*p*=0.9565), 11 Hz (*p*=0.9984), 13 Hz (*p*=0.8630) and 15 Hz (*p*=0.8572) sleep spindles; and P3a amplitude in the difference waveform during mismatch negativity (*p*=0.0683).

Cross-frequency coupling: We found no significant differences in delta phase/gamma power cross-frequency coupling between groups as measured by the MI [7-9] (*Grin2a* mice, 3 months: *p=*0.8998; *Grin2a* mice, 6 months: *p=*0.5117; *Akap11* mice, 3 months: *p=*0.9754; *Akap11* mice, 6 months: *p*=0.7309).

**REFERENCES**

1. McHugh ML. Interrater reliability: the kappa statistic. *Biochemia Medica* 2012; 22(3): 276-282.
2. Ghoshal A, Uygun DS, Yang L, McNally JM, Lopez-Huerta VG, Arias-Garcia MA *et al.* Effects of a patient-derived de novo coding alteration of CACNA1I in mice connect a schizophrenia risk gene with sleep spindle deficits. *Translational Psychiatry* 2020; 10: 29.
3. Purcell SM, Manoach DS, Demanuele C, Cade BE, Mariani S, Cox R *et al*. Characterizing sleep spindles in 11,630 individuals from the National Sleep Research Resource*.* *Nature Communications* 2017; 8: 15930.
4. Kim D, Hwang E, Lee M, Sung H, Choi JH. Characterization of topographically specific sleep spindles in mice. *Sleep* 2015; 38(1): 85-96.
5. O'Donnell BF, Vohs JL, Krishnan GP, Hetrick WP, Morzorati SL. The auditory steady-state response (ASSR): a translational biomarker for schizophrenia*.* *Supplements to Clinical Neurophysiology* 2013; 62: 101-112.
6. Tada M, Kirihara K, Mizutani SD, Uka T, Kunii N, Koshiyama D *et al.* Mismatch negativity (MMN) as a tool for translational investigations into early psychosis: A review. *International Journal of Psychophysiology* 2019; 145: 5-14.
7. Tort ABL, Komorowski R, Eichenbaum H, Kopell N. Measuring Phase-Amplitude Coupling Between Neuronal Oscillations of Different Frequencies. *Journal of Neurophysiology* 104(2): 1195-1210.
8. Lopes-Aguiar C, Ruggiero RN, Rossignoli MT, Esteves IM, Peixoto-Santos JE, Romcy-Pereira RN *et al.* Long-term potentiation prevents ketamine-induced aberrant neurophysiological dynamics in the hippocampus-prefrontal cortex pathway in vivo. *Science Reports* 2020; 10(1): 7167.
9. Guyon N, Zacharias LR, Fermino de Oliveira E, Kim H, Leite JP, Lopes-Aguiar C *et al*. Network Asynchrony Underlying Increased Broadband Gamma Power. *The Journal of Neuroscience* 2021; 41(13): 2944-2963.
10. Donoghue T, Haller M, Peterson EJ, Varma P, Sebastian P, Gao R *et al.* Parameterizing neural power spectra into periodic and aperiodic components. *Nature Neuroscience* 2020; 23: 1655-1665.

**SUPPLEMENTAL TABLES**

| Gamma band (30-50 Hz) | Spectral entropy (bits) | Aperiodic exponent (µV^2^Hz^-1^) | Adjusted power (µV^2^) |
| --- | --- | --- | --- |
| *Grin2a^+/+^* (3 mo) | 6.3278 ± 0.0029 (*n=*12) | 0.7528 ± 0.0481 (*n*=12) | 0.02336 ± 0.00713 (*n=*12) |
| *Grin2a^+/-^* (3 mo) | 6.3329 ± 0.0006 (*n=*12, *p*=0.114) | 0.6372 ± 0.0274 (*n=*12, *p*=0.158) | 0.03175 ± 0.00596 (*n=*12, *p*=0.6514) |
| *Grin2a^-/-^* (3 mo) | 6.3343 ± 0.0006 (*n*=12, *p=*0.0354) | 0.5419 ± 0.0501 (*n=*12, *p*=0.0044) | 0.02542 ± 0.00678 (*n=*12, *p*=0.9743) |
| *Grin2a^+/+^* (6 mo) | 6.3161 ± 0.0083 (*n=*11) | 1.121 ± 0.180 (*n*=11) | 0.0344 ± 0.0111 (*n*=11) |
| *Grin2a^+/-^* (6 mo) | 6.3302 ± 0.0013 (*n=*12, *p*=0.0757) | 0.7478 ± 0.0491 (*n=*12, *p*=0.0383) | 0.0293 ± 0.0110 (*n=*12, *p*=0.9196) |
| *Grin2a^-/-^* (6 mo) | 6.3311 ± 0.0011 (*n=*12, *p*=0.0551) | 0.7196 ± 0.0470 (*n=*12, *p*=0.0244) | 0.0230 ± 0.0043 (*n=*12, *p*=0.6664) |
| *Akap11^+/+^* (3 mo) | 6.3248 ± 0.0011 (*n=*12) | 0.9630 ± 0.0364 (*n*=12) | 0.06375 ± 0.01876 (*n*=12) |
| *Akap11^+/-^* (3 mo) | 6.3256 ± 0.0013 (*n=*12, *p*=0.858) | 0.9495 ± 0.0475 (*n*=12, *p*=0.961) | 0.01508 ± 0.00287 (*n*=12, *p*=0.0261) |
| *Akap11^-/-^* (3 mo) | 6.3366 ± 0.0002 (*n=*12, *p*=4.53e-09) | 0.4483 ± 0.0148 (*n*=12, *p*=2.69e-11) | 0.02900 ± 0.01067 (*n*=12, *p*=0.1398) |
| *Akap11^+/+^* (6 mo) | 6.3191 ± 0.0038 (*n=*12) | 1.111 ± 0.092 (*n*=12) | 0.04133 ± 0.01620 (*n*=12) |
| *Akap11^+/-^* (6 mo) | 6.3170 ± 0.0059 (*n=*12, *p*=0.924) | 1.121 ± 0.118 (*n*=12, *p*=0.997) | 0.07075 ± 0.03740 (*n*=12, *p*=0.6880) |
| *Akap11^-/-^* (6 mo) | 6.3358 ± 0.0008 (*n*=12, *p=*0.0173) | 0.4636 ± 0.0482 (*n*=12, *p*=4.91e-05) | 0.04050 ± 0.01509 (*n*=12, *p*=0.9997) |

**Table S1. Spectral entropy and parameterization of gamma oscillations across genotypes.** Mean ± standard error of the spectral entropy, aperiodic exponent, and aperiodic-adjusted power for the gamma band (30-50 Hz) during NREM sleep are reported for each genotype. Dark blue cells indicate statistically significant differences (*p<*0.05) when compared to the control group (*Grin2a^+/+^* or *Akap11^+/+^*) for that age cohort, while light blue cells indicate a trend approaching significance (*p*<0.10). At 3 and 6 months, *Grin2a* heterozygous and homozygous mutants and *Akap11* homozygous mutants exhibited significantly increased spectral entropy and/or reduced aperiodic exponents in the parameterized power spectra. Conversely, few significant differences were observed in the aperiodic-adjusted gamma power.

|  | Open field locomotion | Sleep patterns | Baseline EEG power | Sleep spindles | Auditory steady-state responses (ASSR) | Mismatch negativity (MMN) |
| --- | --- | --- | --- | --- | --- | --- |
| *Grin2a* mutants | ↑ in KO | Normal | ↑ gamma in Het and KO, ↑ broadband power in KO | ↑ in Het and KO | ↓ 50 Hz ASSR in Het and KO | ↓ P3a, P1 response to deviant stimuli in KO |
| *Akap11* mutants | ↓ in Het and KO | ↓ NREM in KO | ↑ gamma in KO | ↓ in Het and KO | Trend towards ↓ 40 Hz ASSR in KO | ↓ P3a response in difference waveform in KO |

**Table S2. Results summary.** Summary of the major differences found in *Grin2a* and *Akap11* Het and KO mutant mice, as compared to WT littermates.

**SUPPLEMENTAL FIGURES**


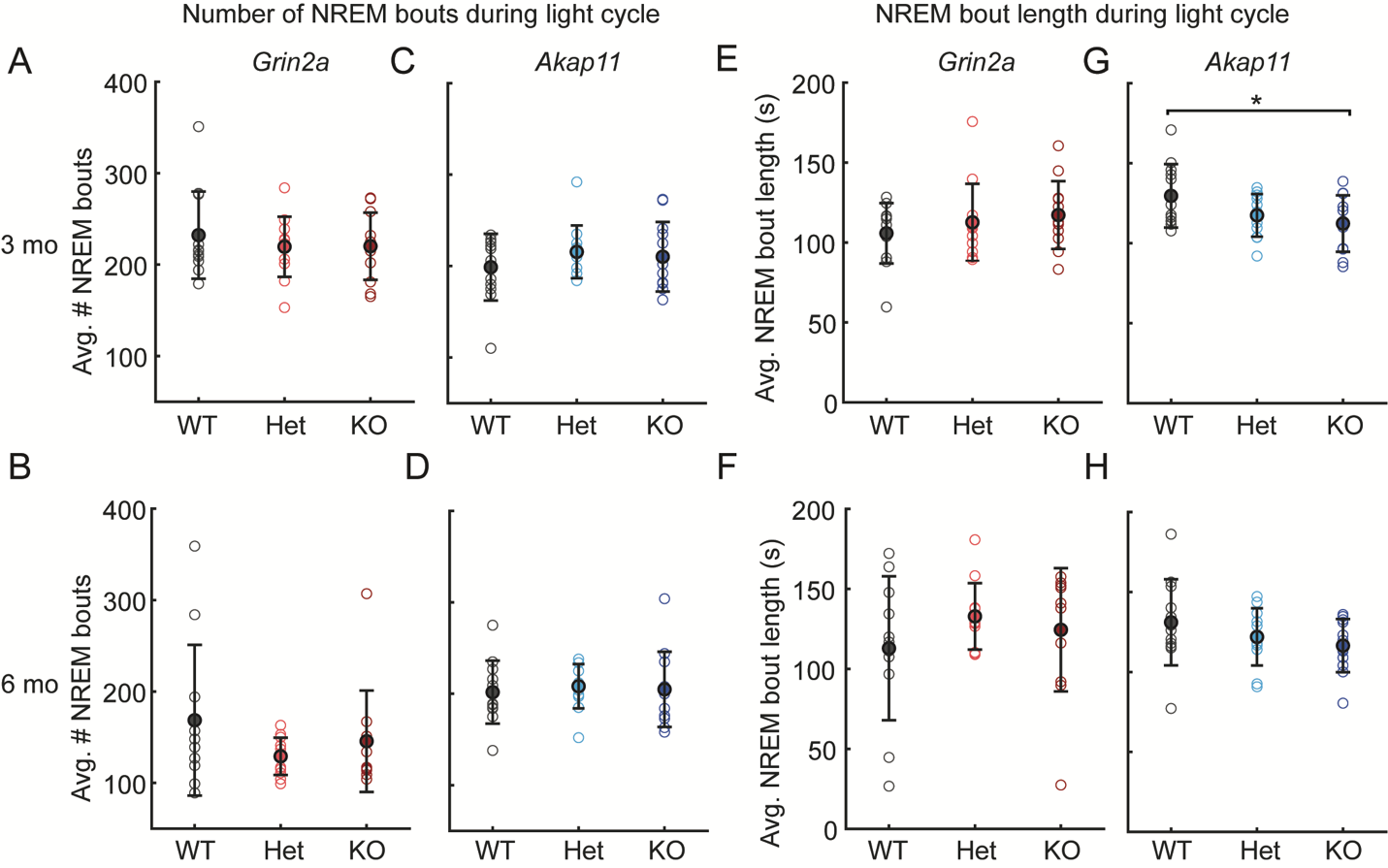


**Figure S1. Sleep fragmentation patterns of *Grin2a* and *Akap11* mutant mice.** **(A-D)** Average number of NREM bouts during the light cycle. *Grin2a* and *Akap11* mutants both exhibited similar bout numbers as their WT littermates, suggesting that the NREM deficiencies found in *Akap11^-/-^* mice (see Figure 2) are not due to sleep fragmentation. **(E-H)** Average NREM bout length during the light cycle. At 3 months, *Akap11^-/-^* mice exhibited shorter NREM bout lengths than their WT littermates (*p*=0.0478). Error bars denote mean ± standard error; *p*<0.05, ***p*<0.01, ****p*<0.001; *n*=11-12 mice/group.

**
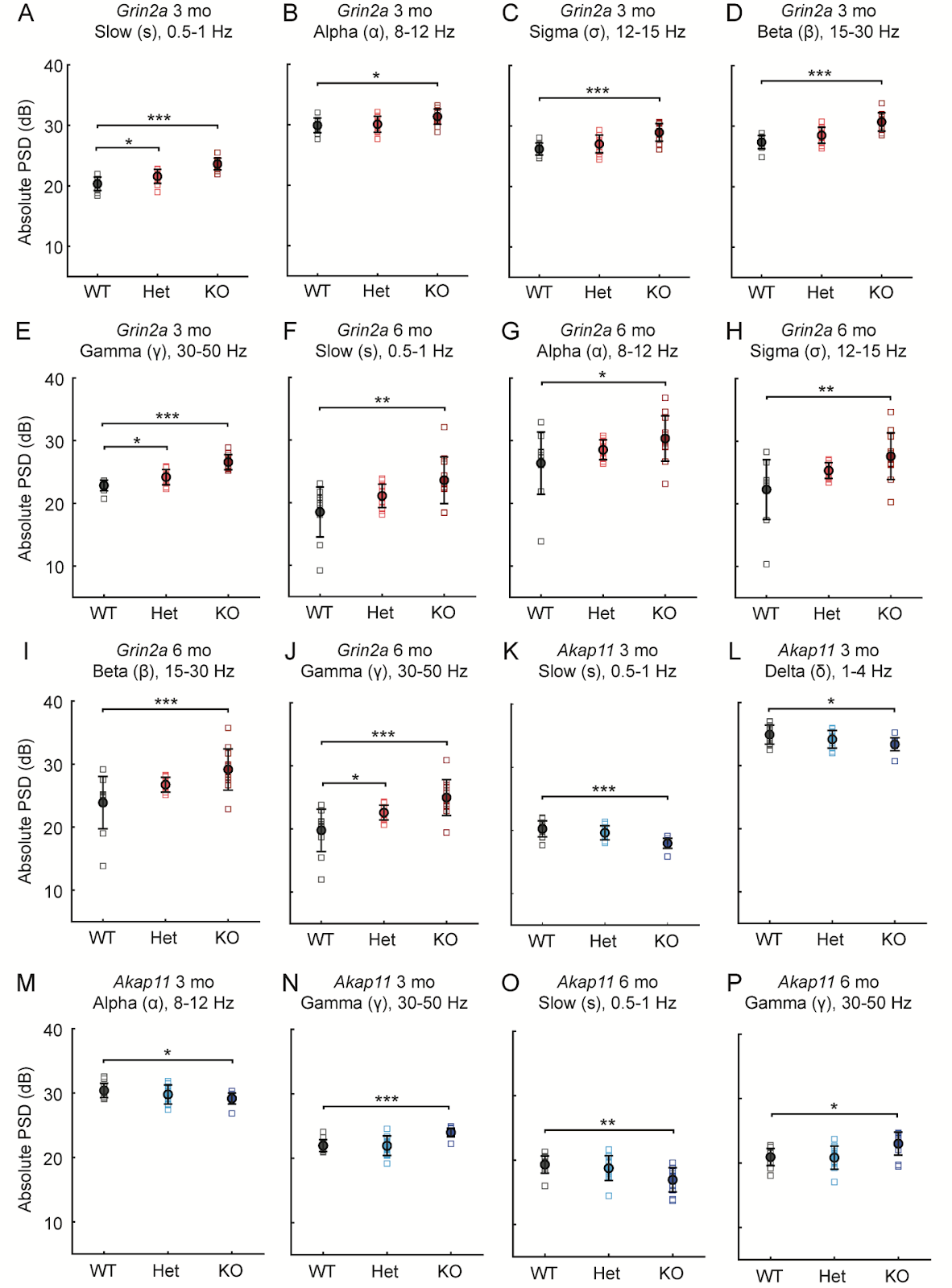
Figure S2. Absolute PSD of brain oscillations in *Grin2a* and *Akap11* mutant mice during NREM sleep.** Absolute power for 3- and 6-month *Grin2a* **(A-J)** and *Akap11* **(K-P)** mutants in NREM sleep during the light cycle. Absolute PSD was computed for each of the following frequency bands: slow (s), 0.5-1 Hz; delta (δ), 1-4 Hz; theta (θ), 4-8 Hz; alpha (α), 8-12 Hz; sigma (σ), 12-15 Hz; beta (β), 15-30 Hz; gamma (γ), 30-50 Hz. Quantification of significant differences (see Figure 3) are shown here. Error bars show mean ± standard error; *p*<0.05, ***p*<0.01, ****p*<0.001; *n*=11-12 mice/group.


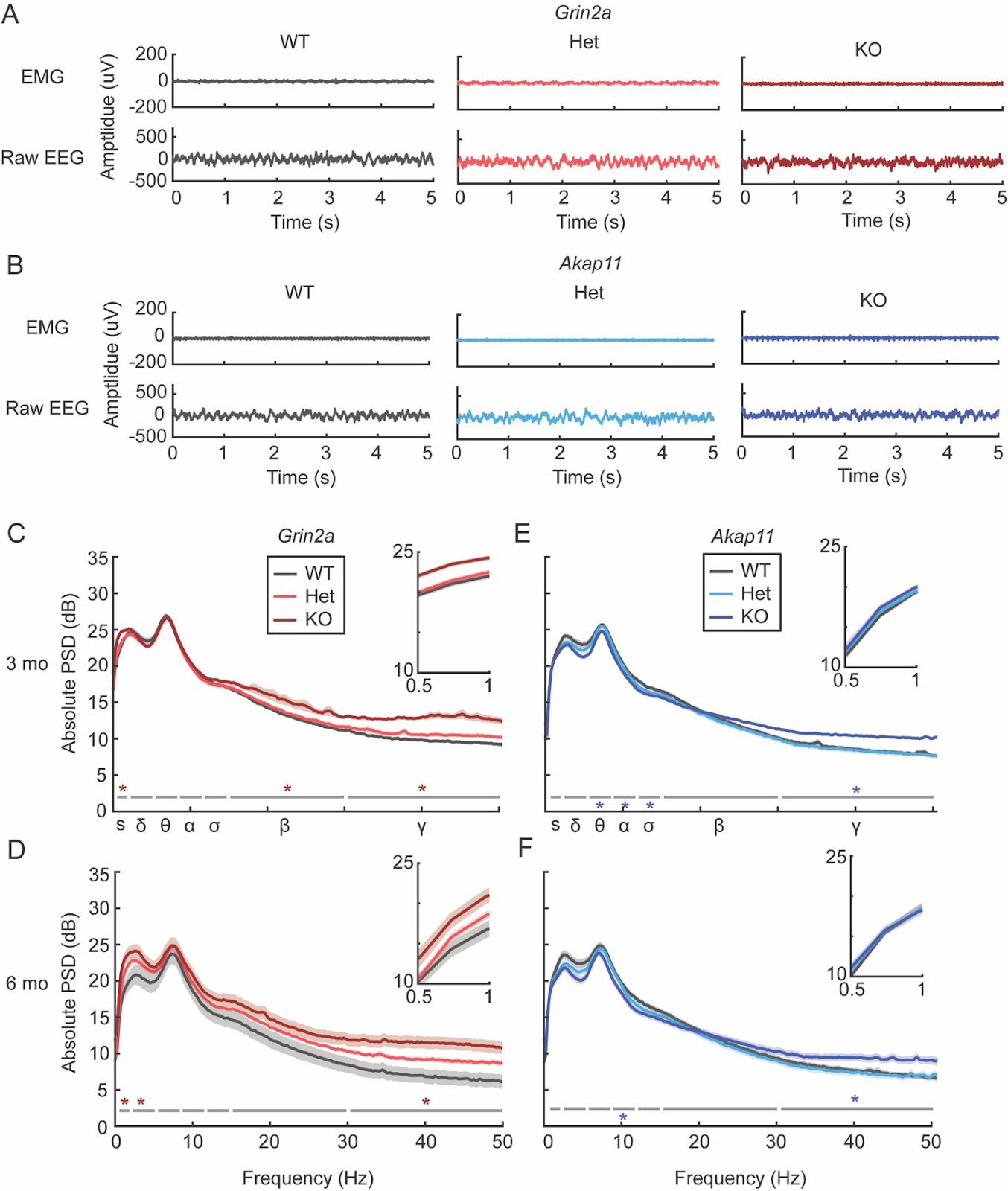


**Figure S3. Power spectral analysis of brain oscillations in *Grin2a* and *Akap11* mutant mice during REM sleep.** **(A-B)** Representative EMG and EEG traces from Grin2a and Akap11 animals during REM sleep. **(C-F)** Absolute REM power spectra for 3- and 6-month *Grin2a* and *Akap11* mutants during the light cycle. Light and dark red/blue stars indicate oscillations for which +/- and -/- mice, respectively, differed significantly from WT littermates (*p*<0.05) in each of the frequency bands: slow (s), 0.5-1 Hz; delta (δ), 1-4 Hz; theta (θ), 4-8 Hz; alpha (α), 8-12 Hz; sigma (σ), 12-15 Hz; beta (β), 15-30 Hz; gamma (γ), 30-50 Hz; with stars above or below the gray line indicating significant increases or decreases in power, respectively. *Grin2a^-/-^* (3 months: *p*=2.99e-07; 6 months: *p*=8.29e-04) and *Akap11^-/-^* (3 months: *p*=2.87e-04; 6 months: *p*=0.0435) mice exhibited increased gamma oscillations, similar to NREM sleep (Figure 3). *Grin2a^-/-^* animals additionally exhibited increases in slow (3 months: *p*=7.95e-07; 6 months: *p*=0.0056), beta (3 months: *p*=0.0195) and delta oscillations (6 months: *p*=0.0406), while *Akap11^-/-^* animals had reduced theta (3 months: *p*=0.0138), alpha (3 months: *p*=0.0036; 6 months: *p*=0.0473) and sigma (3 months: *p*=0.0397) oscillations. Shading indicates mean ± standard error. Insets show magnified view of the graph in the slow oscillation range (0.5-1 Hz). *n*=11-12 mice/group.


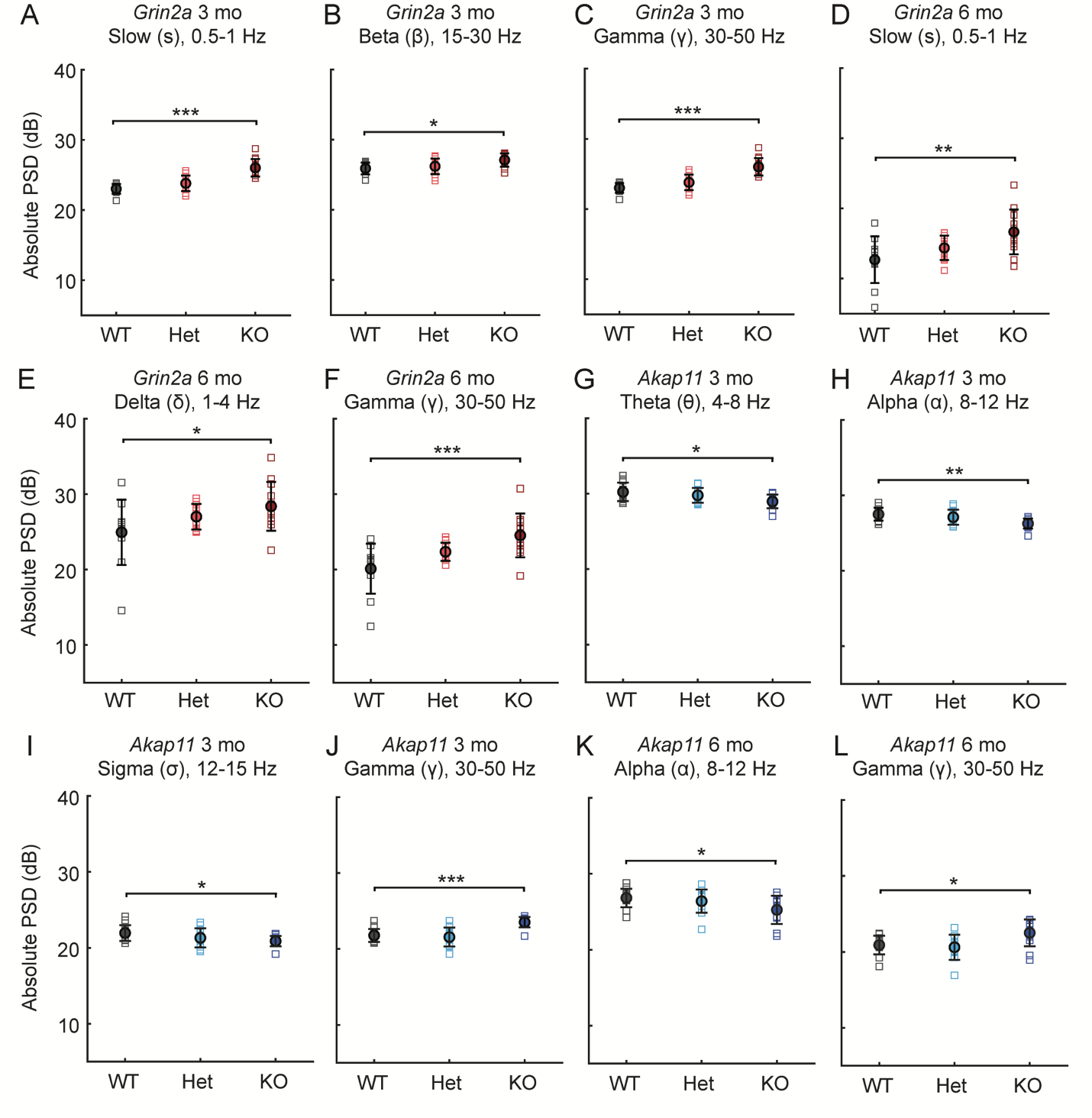


**Figure S4. Absolute PSD of brain oscillations in *Grin2a* and *Akap11* mutant mice during REM sleep**. Absolute power for 3- and 6-month *Grin2a* **(A-J)** and *Akap11* **(K-P)** mutants in REM sleep during the light cycle. Absolute PSD was computed for each of the following frequency bands: slow (s), 0.5-1 Hz; delta (δ), 1-4 Hz; theta (θ), 4-8 Hz; alpha (α), 8-12 Hz; sigma (σ), 12-15 Hz; beta (β), 15-30 Hz; gamma (γ), 30-50 Hz. Quantification of significant differences (see Figure S3) are shown here. Error bars show mean ± standard error; *p*<0.05, ***p*<0.01, ****p*<0.001; *n*=11-12 mice/group.


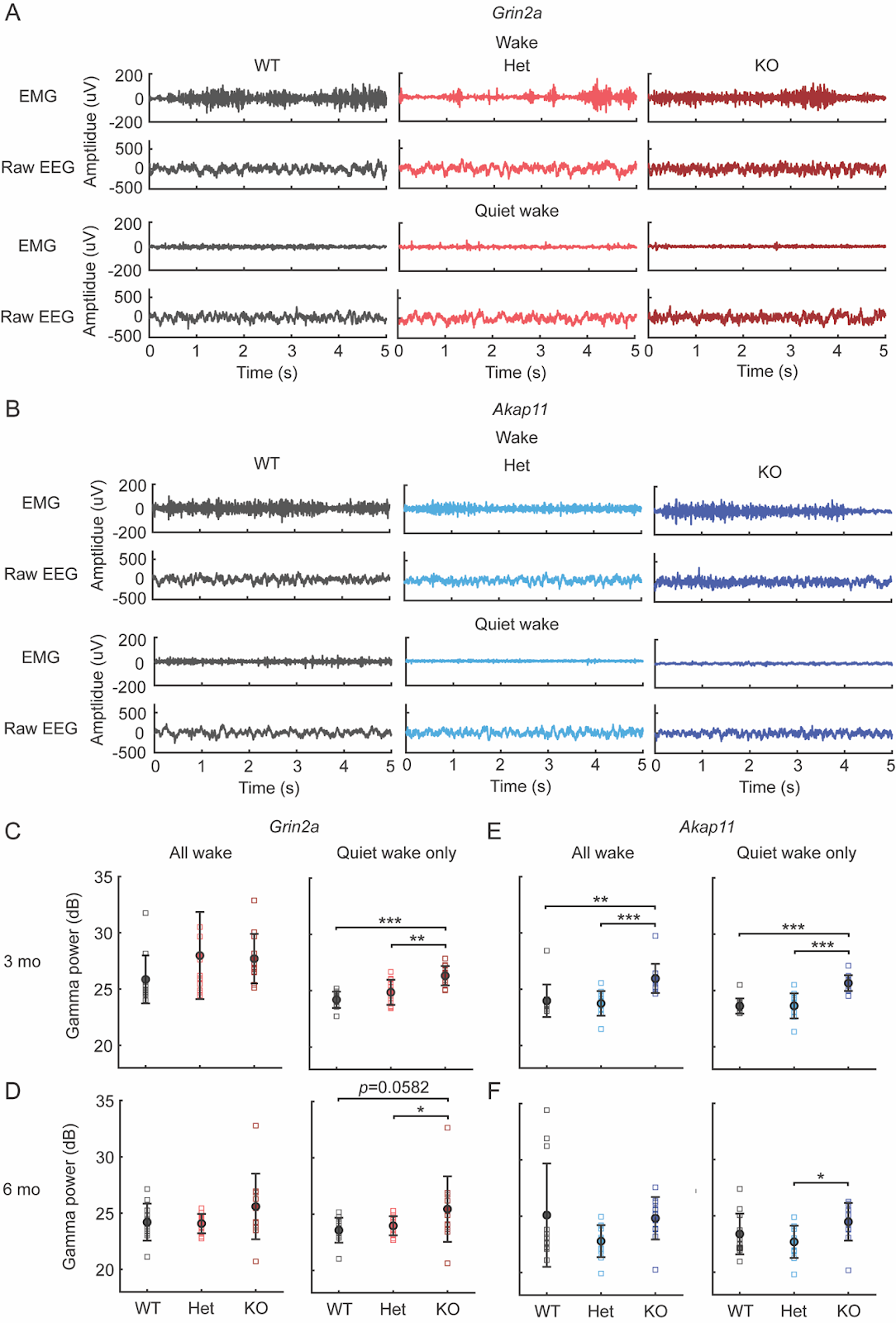


**Figure S5. Gamma oscillations in *Grin2a* and *Akap11* mutant mice during wake.** **(A-B)** Representative EMG and EEG traces for *Grin2a* and *Akap11* mutants during wake and quiet wake (periods of presumed immobility during wakefulness; see Supplementary Methods for more details). **(C-F)** Absolute PSD for 3- and 6-month *Grin2a* **(C-D)** and *Akap11* **(E-F)** mutants computed for gamma (γ), 30-50 Hz, for wake states during the dark cycle. During wake, *Akap11^-/-^* animals showed increased gamma power compared to WT littermates at 3 months (*p*=5.06e-04), with heterozygous animals similar to WT. Analysis of quiet wake states revealed additional increases in gamma power in *Grin2a^-/-^* animals at 3 months (*p*=6.87e-06) and a trend towards increased gamma in KO at 6 months (*p*=0.0582), similar to NREM (Figure 3) and REM sleep (Figure S3-4). Both *Grin2a* and *Akap11* heterozygous mutants exhibited similar gamma power as WT littermates. Error bars show mean ± standard error; **p*<0.05, ***p*<0.01, ****p*<0.001; *n*=11-12 mice/group.

**
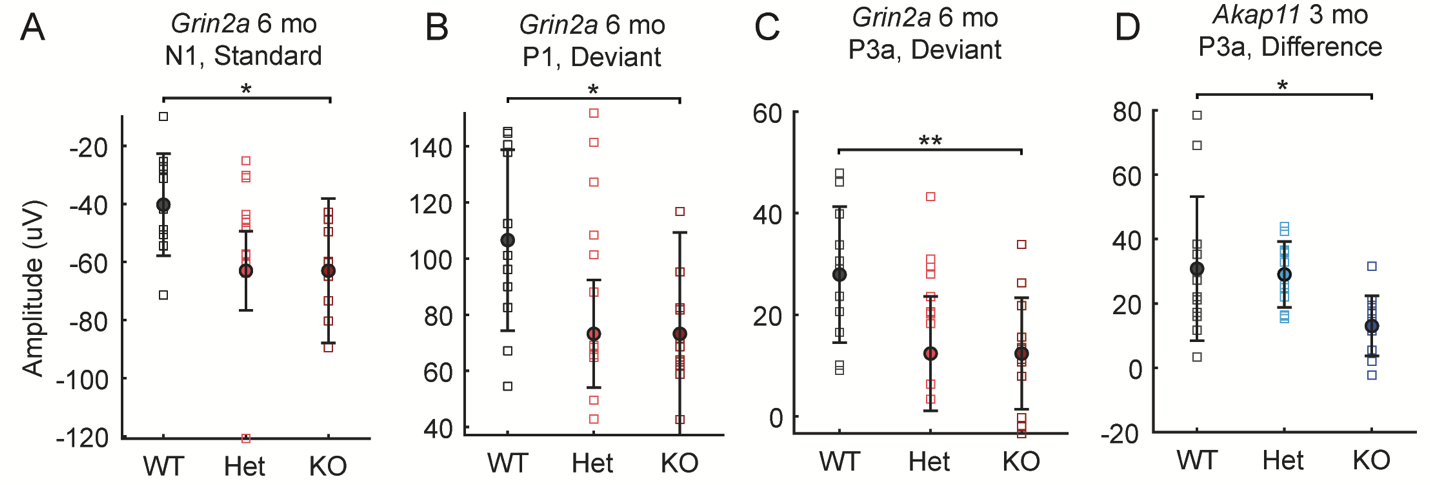
Figure S6. Peak amplitudes during the mismatch negativity test in *Grin2a* and *Akap11* mutants.** Quantification of peak amplitudes for event-related potential components in the mismatch negativity test (Figure 6); significant results are shown for **A)** *Grin2a*, 6 months, N1 response to standard tones; **B)** *Grin2a*, 6 months, P1 and **C)** P3a response to deviant tones; **D)** *Akap11*, 3 months, P3a response in difference waveform [the response to deviant tones minus the response to standard tones]. Error bars indicate ± standard error; *p*<0.05, ***p*<0.01; *n*=11-12 mice/group.
